# Supplementary material for: MetaQTL: a package of new computational methods for the meta-analysis of QTL mapping experiments
Source: BMC Bioinformatics. 2007 Feb 8;8:49. doi: 10.1186/1471-2105-8-49 (PMC1808479; doi:10.1186/1471-2105-8-49)
Supplement: Additional File 7 — MetaQTL Package : jar file and tutorial. This Zip archive contains both the MetaQTL JAR file and the files of the tutorial. [file 1471-2105-8-49-S7.zip › org.inra.metaqtl/doc/org/metaqtl/graph/QtlShape.html]

QtlShape


|  |  |  |
| --- | --- | --- |
| **Overview** \| **Package** \| **Class** \| **Use** \| **Tree** \| **Deprecated** \| **Index** \| **Help** | |  |
| **PREV CLASS**   **NEXT CLASS** | **FRAMES**    **NO FRAMES**     **All Classes** |  |
| SUMMARY: NESTED \| FIELD \| CONSTR \| METHOD | DETAIL: FIELD \| CONSTR \| METHOD |  |


---


## org.metaqtl.graph Class QtlShape

```
java.lang.Object
  org.metaqtl.graph.GraphShape
      org.metaqtl.graph.QtlShape
```

---

``` public class QtlShape extends GraphShape ```

---

| **Field Summary** | |
| --- | --- |
| `double` | `ci_height` |
| `double` | `ci_width` |
| `double` | `y_from` |
| `double` | `y_pos` |

**Fields inherited from class org.metaqtl.graph.GraphShape**

`height, width, x, y`


**Constructor Summary**

`QtlShape()`


| **Method Summary** | |
| --- | --- |
| `void` | `buildShape(java.awt.Graphics2D graph, java.lang.Object object)`             This methods create a new QtlShape from the given graphics context. |
| `void` | `draw(java.awt.Graphics2D graph)`             Draws the shape. |
| `double` | `getHeigth()` |
| `double` | `getWidth()` |
| `static double` | `getYDistance(QtlShape s1, QtlShape s2)`             Computes the distance between two Qtl shapes assuming along the same axe Y (i.e the shapes have same X). |
| `double` | `getYMax()` |
| `double` | `getYMin()` |

**Methods inherited from class org.metaqtl.graph.GraphShape**

`getHeight`

**Methods inherited from class java.lang.Object**

`clone, equals, finalize, getClass, hashCode, notify, notifyAll, toString, wait, wait, wait`

**Field Detail**

### y\_pos

```
public double y_pos
```

---


### y\_from

```
public double y_from
```

---


### ci\_height

```
public double ci_height
```

---


### ci\_width

```
public double ci_width
```


**Constructor Detail**

### QtlShape

```
public QtlShape()
```


**Method Detail**

### buildShape

```
public void buildShape(java.awt.Graphics2D graph,
                       java.lang.Object object)
```

:   This methods create a new QtlShape from the given
    graphics context.

    :   **Specified by:**: `buildShape` in class `GraphShape`
    :   **Parameters:**: `graph` -: `font` -: `unit` -

---


### draw

```
public void draw(java.awt.Graphics2D graph)
```

:   **Description copied from class: `GraphShape`**
:   Draws the shape.

    :   **Specified by:**: `draw` in class `GraphShape`

---


### getHeigth

```
public double getHeigth()
```

---


### getWidth

```
public double getWidth()
```

:   **Overrides:**: `getWidth` in class `GraphShape`

---


### getYMin

```
public double getYMin()
```

:   **Specified by:**: `getYMin` in class `GraphShape`

---


### getYMax

```
public double getYMax()
```

:   **Specified by:**: `getYMax` in class `GraphShape`

---


### getYDistance

```
public static double getYDistance(QtlShape s1,
                                  QtlShape s2)
```

:   Computes the distance between two Qtl shapes assuming
    along the same axe Y (i.e the shapes have same X).

    :   **Parameters:**: `shape` -: `prev` - **Returns:**


---


|  |  |  |
| --- | --- | --- |
| **Overview** \| **Package** \| **Class** \| **Use** \| **Tree** \| **Deprecated** \| **Index** \| **Help** | |  |
| **PREV CLASS**   **NEXT CLASS** | **FRAMES**    **NO FRAMES**     **All Classes** |  |
| SUMMARY: NESTED \| FIELD \| CONSTR \| METHOD | DETAIL: FIELD \| CONSTR \| METHOD |  |


---
